# CONTEXT MATTERS IN DECISION-MAKING: CAREGIVING NETWORKS OF AFRICAN AMERICAN DEMENTIA DYADS

**DOI:** 10.1093/geroni/igac059.778

**Published:** 2022-12-20

**Authors:** Kalisha Bonds Johnson, Fayron Epps, MinKyoung Song, Karen Lyons, Kenneth Hepburn, Martha Driessnack

**Affiliations:** Emory University, Atlanta, Georgia, United States; Emory University, Fairburn, Georgia, United States; Oregon Health & Science University, Portland, Oregon, United States; Boston College, Chestnut Hill, Massachusetts, United States; Emory University, Atlanta, Georgia, United States; Oregon Health & Science University, Portland, Oregon, United States

## Abstract

Limited research explores the contextual and cultural nuances within African American dementia dyads within the United States and how these factors influence decision-making processes. Through a secondary data analysis of semi-structured interviews, we examined decision-making processes in five African American dementia dyads related to how they navigated decisions for the person living with Alzheimer’s disease and related dementias (ADRD) across five unique contexts (e.g., mother with multiple daughters, mother with son and daughter where the son was the “primary” caregiver). Analysis revealed that within dyads, persons living with ADRD were involved in the decision-making processes, but the level of the involvement in decision making by the caregiving networks varied across dyads. Understanding the context in which decisions are made (i.e., within the dyad, across multiple family members in a caregiving network) has important implications in clinical practice and research. Interventions should be tailored to reflect these contextual and cultural nuances.

